# Assessment of Cesarean Delivery and Neurodevelopmental and Psychiatric Disorders in the Children of a Population-Based Swedish Birth Cohort

**DOI:** 10.1001/jamanetworkopen.2021.0837

**Published:** 2021-03-05

**Authors:** Tianyang Zhang, Gustaf Brander, Ängla Mantel, Ralf Kuja-Halkola, Olof Stephansson, Zheng Chang, Henrik Larsson, David Mataix-Cols, Lorena Fernández de la Cruz

**Affiliations:** 1 Department of Clinical Neuroscience, Centre for Psychiatry Research, Karolinska Institutet, Stockholm, Sweden; 2 Stockholm Health Care Services, Stockholm County Council, Stockholm, Sweden; 3 Department of Medical Epidemiology and Biostatistics, Karolinska Institutet, Stockholm, Sweden; 4 Department of Medical Biochemistry and Microbiology, Science for Life Laboratory, Uppsala University, Uppsala, Sweden; 5 Department of Women’s Health, Karolinska University Hospital, Stockholm, Sweden; 6 Clinical Epidemiology Division, Department of Medicine Solna, Karolinska Institutet, Stockholm, Sweden; 7 School of Medical Sciences, Örebro University, Örebro, Sweden

## Abstract

**Importance:**

Recent studies suggest that cesarean delivery (CD) is associated with increased risk of neurodevelopmental disorders in children, although they were unable to control for indications for CD or familial confounding beyond full siblings.

**Objective:**

To examine the association between CD and neurodevelopmental and psychiatric disorders in children.

**Design, Setting, and Participants:**

This Swedish register-based cohort study included 1 179 341 term-birth singletons born between January 1, 1990, and December 31, 2003, and followed up through December 31, 2013. All individuals were linked to their full siblings, maternal and paternal half siblings, and maternal full cousins. Statistical analyses were performed from September 26, 2019, to January 16, 2021.

**Exposures:**

Birth by CD recorded at birth, stratified into planned and intrapartum CD.

**Main Outcomes and Measures:**

Registered diagnoses of neurodevelopmental disorders, including attention-deficit/hyperactivity disorder (ADHD), autism spectrum disorders (ASD), intellectual disability, tic disorders, communication disorders, learning disorders, and any neurodevelopmental disorder; and psychiatric disorders, including anxiety disorders, obsessive-compulsive disorder, depressive disorders, eating disorders, bipolar disorders, psychotic disorders, and any psychiatric disorder.

**Results:**

Of 1 179 341 individuals, 1 048 838 (533 140 boys [50.8%]) were delivered vaginally, 59 514 (30 138 boys [50.6%]) were delived via planned CD, and 70 989 (39 191 boys [55.2%]) were delivered via intrapartum CD. Mean (SD) age at follow-up was 17.7 (4.1) years for vaginal delivery, 16.6 (4.2) years for planned CD, and 16.8 (4.1) years for intrapartum CD. Compared with vaginal delivery, and after controlling for measured covariates (parental and neonatal characteristics, maternal comorbidities, and pregnancy complications), CD was associated with higher risk in children of any neurodevelopmental disorder (planned CD, hazard ratio [HR], 1.17; 95% CI, 1.13-1.22; intrapartum CD, HR, 1.10; 95% CI, 1.05-1.14), ADHD (planned CD, HR, 1.17; 95% CI, 1.12-1.23; intrapartum CD, HR, 1.10; 95% CI, 1.05-1.15), and intellectual disability (planned CD, HR, 1.26; 95% CI, 1.14-1.39; intrapartum CD, HR, 1.17; 95% CI, 1.06-1.28). Only planned CD was associated with a higher risk of ASD (HR, 1.20; 95% CI, 1.10-1.31), communication disorders (HR, 1.14; 95% CI, 1.02-1.28), and learning disorders (HR, 1.15; 95% CI, 1.01-1.30). Cesarean delivery was not associated with the remaining disorders. The associations between CD and any neurodevelopmental disorder, ADHD, ASD, and intellectual disability attenuated in full cousins and paternal half siblings, and further attenuated (became nonsignificant) in maternal half siblings and full siblings (risk of any neurodevelopmental disorder in full siblings, planned CD, HR, 0.93; 95% CI, 0.81-1.06; intrapartum CD, HR, 1.07; 95% CI, 0.96-1.21).

**Conclusions and Relevance:**

The findings of this study suggest that the association between CD and increased risk of neurodevelopmental disorders in the children was most likely explained by unmeasured familial confounding.

## Key Points

**Question:**

Is cesarean delivery associated with neurodevelopmental and psychiatric disorders in children?

**Findings:**

In this Swedish population-based birth cohort study of more 1.1 million children, births via planned or intrapartum cesarean delivery were associated with a moderately increased risk of neurodevelopmental disorders in children, but these associations were attenuated after adjusting for familial factors.

**Meaning:**

The association between cesarean delivery and neurodevelopmental disorders may be explained by unmeasured familial confounding; observational studies on the association between prenatal risk factors and child outcomes may be spurious owing to unmeasured confounders.

## Teaser

This cohort study uses data from multiple Swedish birth registries to assess whether cesarean birth is associated with neurodevelopmental or psychiatric disorders in children delivered in this manner.

## Introduction

Neurodevelopmental disorders are a group of childhood-onset disorders that often persist into adulthood.^1^ Family studies suggest that these disorders are familial and highly heritable,^2,3,4,5,6,7^ although their specific etiology remains unknown.^8^ Birth by cesarean delivery (CD) has been suggested to represent an environmental risk factor for neurodevelopmental and psychiatric disorders. Previous meta-analyses suggest that birth by CD is associated with increased risk of autism spectrum disorders (ASD) and attention-deficit/hyperactivity disorder (ADHD).^9,10^ However, the mechanisms behind these associations remain poorly understood, and, as with most observational studies, causality is often questioned because of the inability to rule out confounding. Some researchers have theorized that children delivered via CD may bypass the birth canal and acquire and colonize the first microbiota from the hospital environment rather than from their mothers.^11,12,13,14^ Consequently, the altered microbiome composition at birth may lead to disturbed child development, including brain development via the gut-brain axis.^15^ Additionally, intrapartum CD is often the result of complications during pregnancy (eg, preeclampsia) or delivery (eg, fetal distress), which could affect brain development. Previous studies have suggested that the described associations between CD and ASD or ADHD may not be causal but due to familial confounding.^16,17,18,19^ Furthermore, a potential genetic overlap between psychiatric disorders and the likelihood of delivering via CD has been suggested,^20,21^ indicating that CD may reflect the child’s genetic predisposition to develop psychiatric disorders rather than being a causal risk factor.

In this longitudinal, population-based cohort study, we aimed to thoroughly examine the association between planned or intrapartum CD and risk of neurodevelopmental and psychiatric disorders in children. We distinguished planned and intrapartum CD because they generally have different clinical indications and may be associated with different levels of exposure to maternal gut microbiota.^22^ We also aimed to explore whether indications for CD contributed to the association. To explore whether the association can be further explained by familial confounders, we repeated the analysis in clusters of relatives with different degrees of relatedness.

## Methods

Ethical approval for this cohort study was obtained from the Regional Ethical Review Board in Stockholm. The requirement for informed consent was waived because the study was register-based and data on the included individuals were deidentified. This study complies with the Strengthening the Reporting of Observational Studies in Epidemiology (STROBE) reporting guideline.

### Study Population

**Figure 1.**
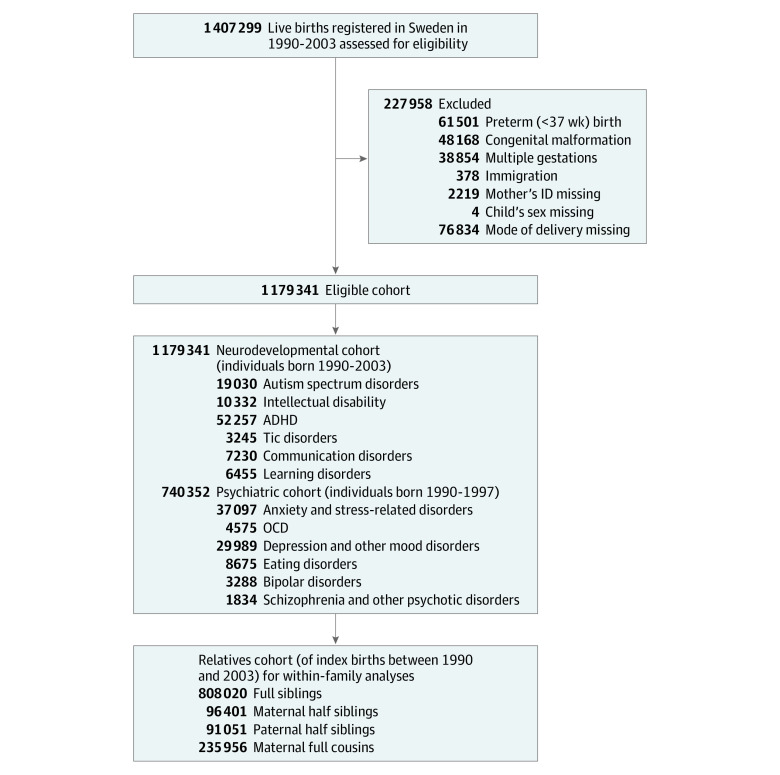
Study Population ADHD indicates attention-deficit/hyperactivity disorder; ID, identification number; OCD, obsessive-compulsive disorder.

Data were obtained by linking individuals through their unique personal identification number^23^ from multiple Swedish registries, including (1) the Medical Birth Register,^24,25^ (2) the Multi-Generation Register,^26^ (3) the National Patient Register,^27^ (4) the Prescribed Drug Register,^28^ (5) the Longitudinal Integrated Database for Health Insurance and Labour Studies,^29^ (6) the Total Population Register,^30^ and (7) the Cause of Death Register^31^ (eMethods 1 in the Supplement). The study population is described in Figure 1. First, we identified all 1 407 299 live births in Sweden between January 1, 1990, and December 31, 2003. After applying the exclusion criteria, 1 179 341 individuals remained and were followed up from birth until the first diagnosis (differing between the outcomes), emigration, death, or December 31, 2013, whichever came first. For some outcomes (ie, psychiatric disorders) that tend to have a later onset, we trimmed the main cohort to births between January 1, 1990, and December 31, 1997, to allow for longer follow-ups (Figure 1). For the within-family analyses, we identified full siblings, maternal half siblings, paternal half siblings, and maternal full cousins (Figure 1).

### Outcomes

We included a list of disorders of relatively high prevalence and, for practical reasons, based on their typical age at onset, grouped them into neurodevelopmental disorders or psychiatric disorders (eTable 1 in the Supplement). Neurodevelopmental disorders, which tend to have an early onset, included ADHD, ASD, intellectual disability, and tic, communication, and learning disorders. For ADHD, cases were additionally ascertained by the dispensing of ADHD drugs.^32^ Additionally, we computed a composite any developmental disorder outcome, which refers to having at least 1 of the neurodevelopmental disorders mentioned previously.

Psychiatric disorders included anxiety and stress-related disorders, obsessive-compulsive disorder, depression and other mood disorders, eating disorders, bipolar disorders, and schizophrenia and other psychotic disorders. A composite any psychiatric disorder outcome was computed, which refers to having at least 1 of the psychiatric disorders mentioned previously. We identified cases using the first instance of a recorded *International Classification of Diseases, Ninth Revision (ICD-9)* or *International Statistical Classification of Diseases and Related Health Problems, Tenth Revision (ICD-10)* diagnosis in the National Patient Register.

### Exposures and Covariates

Information on mode of delivery has been recorded in the Medical Birth Register since 1973, but information on whether a CD was performed before or after labor onset (classified into planned or intrapartum CD) was included in the register records only after 1990. Hence, 1990 marked the starting year of our cohort. Vaginal delivery included unassisted and assisted (use of forceps or vacuum extraction) vaginal births. Covariates included (1) maternal characteristics and pregnancy complications, including age at delivery, parity, highest education level at delivery, smoking during pregnancy, hypertension, diabetes, infections during pregnancy, polyhydramnios, oligohydramnios, preeclampsia or eclampsia, pelvic disproportion, placental disorders, and psychiatric history; (2) paternal age at delivery and paternal psychiatric history; (3) birth complications (failed induction, fetal distress, dystocia, and labor malpresentation); and (4) neonatal characteristics (birth year, sex, gestational age, small or large for gestational age birth) (eMethods 2 in the Supplement).

### Statistical Analysis

**Figure 2.**
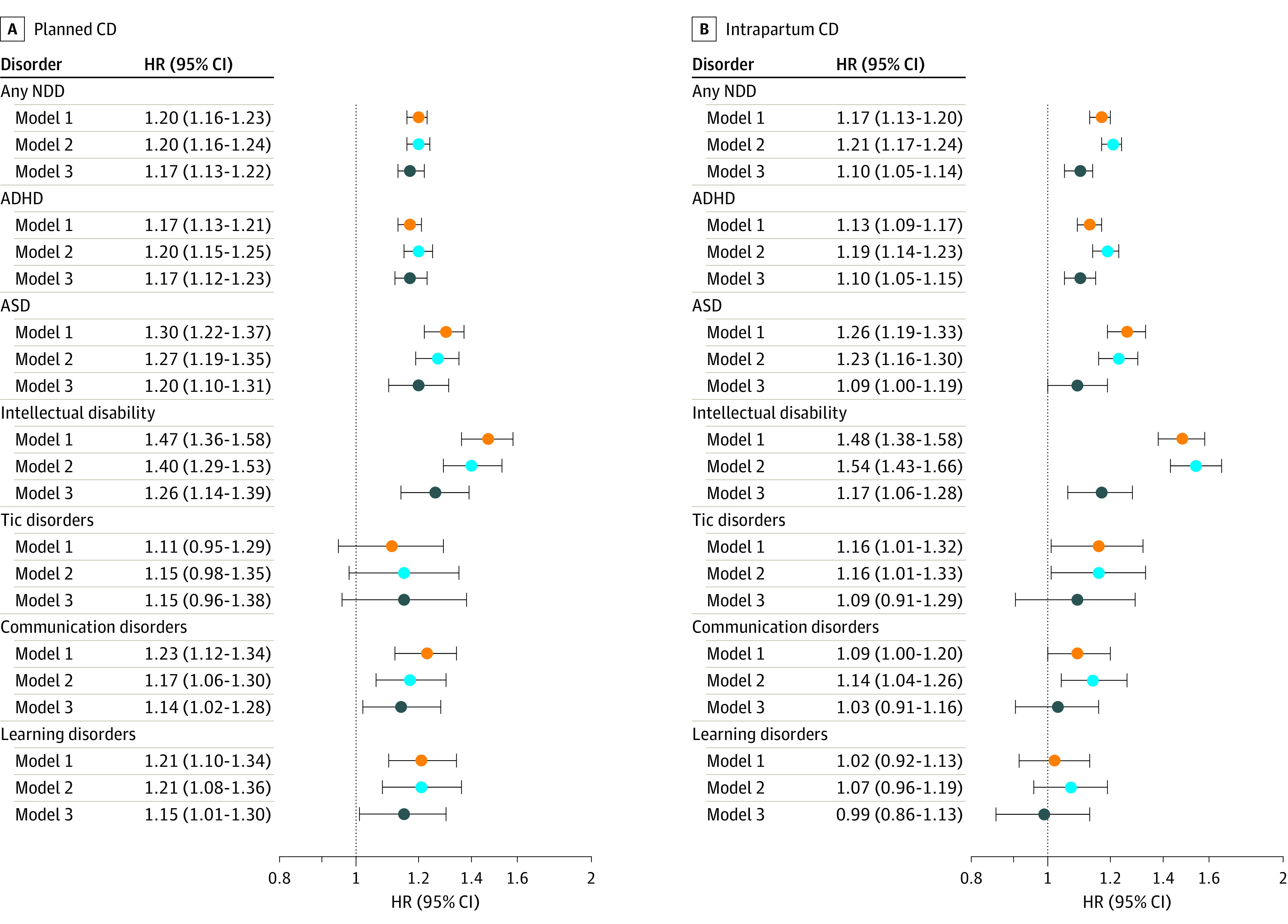
Risk of Neurodevelopmental Disorders (NDD) Among Individuals Delivered by Planned and Intrapartum Cesarean Delivery (CD), Compared With Vaginally Delivered Individuals Hazard ratios (HRs) with 95% CIs are presented with 3 levels of adjustments for each outcome for planned CD (A) and intrapartum CD (B). The outcomes were any neurodevelopmental disorder, including attention-deficit/hyperactivity disorder (ADHD), autism spectrum disorders (ASD), intellectual disability, tic disorders, communication disorders, and learning disorders. Model 1 (orange) adjusted for child’s sex and year of birth. Model 2 (light blue) adjusted for child’s sex and year of birth, gestational age, age of mother and father at birth, parity, mother’s highest education level at birth, maternal smoking during pregnancy, and maternal and paternal history of psychiatric disorders. In addition to variables adjusted in Model 2, Model 3 (dark blue) further adjusted for maternal hypertension, maternal diabetes, maternal infections during pregnancy, fetal malpresentation, large for gestational age, polyhydramnios, oligohydramnios, and preeclampsia. Model 3 included data from panels A and B. For planned CD, we further adjusted for pelvic disproportion. For intrapartum CD, we further adjusted for pelvic disproportion, placenta disorders, dystocia, failed induction, and fetal distress.

**Figure 3.**
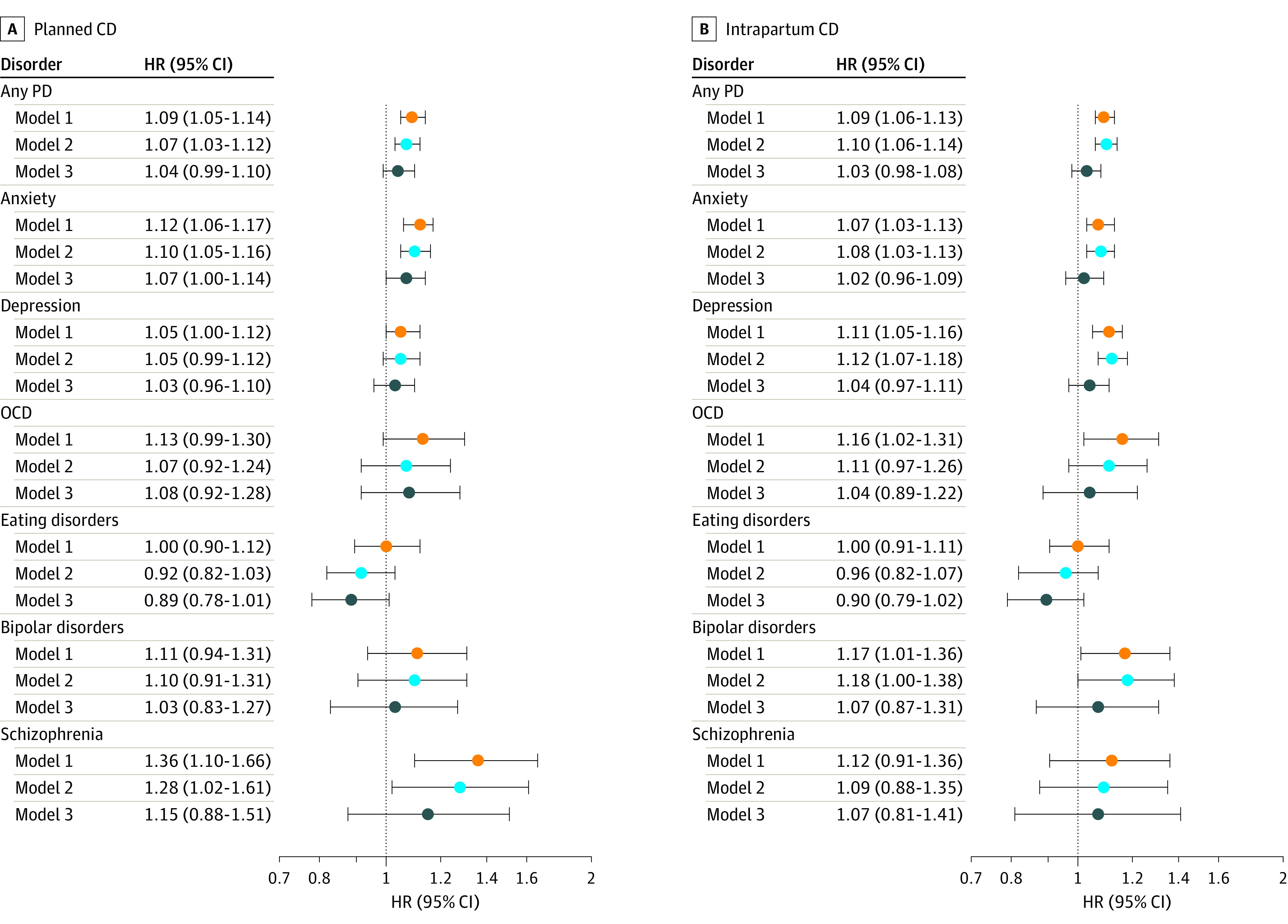
Risk of Psychiatric Disorders (PD) Among Individuals Delivered by Planned and Intrapartum Cesarean Delivery (CD), Compared With Vaginally Delivered Individuals Hazard ratios (HRs) with 95% CIs are presented with 3 levels of adjustments for each outcome for planned CD (A) and intrapartum CD (B). Model 1 (orange) adjusted for children’s sex and year of birth. Outcomes were any PD, anxiety and stress-related disorders, obsessive-compulsive disorder (OCD), depression and other mood disorders; eating disorders, bipolar disorders, and schizophrenia and other psychotic disorders. Model 2 (light blue) adjusted for children’s sex and year of birth, gestational age, age of mother and father at birth, parity, mother’s highest education level at birth, maternal smoking during pregnancy, and maternal and paternal history of psychiatric disorders. In addition to variables adjusted in Model 2, Model 3 (dark blue) further adjusted for maternal hypertension, maternal diabetes, maternal infections during pregnancy, fetal malpresentation, large for gestational age, polyhydramnios, oligohydramnios, and preeclampsia. Model 3 included data from panels A and B. For planned CD, we further adjusted for pelvic disproportion. For intrapartum CD, we further adjusted for pelvic disproportion, placenta disorders, dystocia, failed induction, and fetal distress.

Differences in descriptive, categorical variables were determined using χ^2^ tests. Cox proportional hazards regression analyses were used to estimate hazard ratios (HRs) and 95% CIs for the associations between planned or intrapartum CD and each outcome of interest in the children, with attained age as the underlying time scale. We fitted 3 levels of confounding adjustment strategies (Figure 2 and Figure 3): Model 1 adjusted for child sex and birth year to obtain a baseline estimation; model 2 further adjusted for parental characteristics and gestational age; and model 3 additionally adjusted for specific indications for planned or intrapartum CD.

To assess the robustness of the results, we undertook 2 sensitivity analyses. First, we applied restrictions to yield a more homogeneous population with the following characteristics: mothers’ age at delivery was 20 to 35 years; parity less than 3; no reported smoking during pregnancy; no recorded hypertension, diabetes, or psychiatric disorders; no recorded pregnancy complications; no birth complications; and fetuses were in the normal position or presentation with normal weight for gestational age. Owing to power issues, we only performed this analysis for selected outcomes, including any neurodevelopmental disorder, ADHD, ASD, and intellectual disability. We selected these outcomes because we wanted to further explore the association between CD and any outcome that still had a significant association after adjusting for all measured confounders in the previous analyses. Second, we repeated the main analyses comparing CD with only unassisted vaginal delivery (excluding assisted vaginal delivery).

To explore the potential association of familial genetic and environmental factors, we performed stratified Cox proportional hazards regression models in 4 subsamples of clusters of full siblings, maternal and paternal half siblings, and maternal full cousins. By design, these models accounted for, on average, 50%, 25%, 25%, and 12.5% of the children’s genetic background, respectively. Additionally, because CD is also a maternal trait, it adjusts for 100%, 100%, 0%, and 50% of the mother’s genetic background, respectively. Further, these models adjust for unmeasured shared environment (eg, siblings share more environment than cousins). In each cluster, we adjusted for variables that typically vary between siblings or cousins. Additionally, we adjusted for birth order in full and maternal half-sibling models to address bias from carry-over effects on exposure status.^33^ To understand whether mothers who deliver via CD are less likely to have more children, which in turn may influence the probability of being eligible for the sibling models, we calculated the probability of having a second child by the delivery mode of the first child. Because of power issues, we only performed within-family comparisons for any neurodevelopmental disorder, ADHD, ASD, and intellectual disability. Statistical analyses were conducted from September 26, 2019, to January 16, 2021, using SAS software, version 9.4 (SAS Institute) and R, version 3.6.2 (R Foundation).* P* < .05 was considered statistically significant, and all tests were 2-tailed.

## Results

### Descriptive Statistics

Study characteristics of the main cohort are presented in Table 1. Of 1 179 341 individuals, 1 048 838 (533 140 boys [50.8%]) were delivered vaginally, 59 514 (30 138 boys [50.6%]) were delivered via planned CD, and 70 989 (39 191 boys [55.2%]) were delivered via intrapartum CD. Mean (SD) age at follow-up was 17.7 (4.1) years for vaginal delivery, 16.6 (4.2) years for planned CD, and 16.8 (4.1) years for intrapartum CD. Data on first recorded diagnosis for each disorder are reported in eTable 2 in the Supplement.

**Table 1.** Parental, Perinatal, and Neonatal Characteristics and Neurodevelopmental and Psychiatric Disorders of the Cohort Born in Sweden From 1990 to 2003, by Obstetric Mode of Delivery

Variable^a^	Mode of delivery, No. (%)		
	Vaginal delivery (n = 1 048 838)	CD	
		Planned (n = 59 514)	Intrapartum (n = 70 989)
**Parental characteristics**			
Maternal age, y^b^			
<20	23 693 (2.3)	511 (0.9)	1130 (1.6)
20-29	578 341 (55.1)	2222 (37.3)	34 416 (48.5)
30-39	426 603 (40.7)	33 468 (56.2)	33 088 (46.6)
40	20 201 (1.9)	3315 (5.6)	2355 (3.3)
Maternal education, y^b^			
≤9	91 982 (8.8)	5412 (9.1)	6012 (8.5)
10-12	493 895 (43.1)	27 719 (46.6)	33 010 (46.5)
>12	429 131 (45.0)	24 335 (40.9)	29 420 (41.4)
Missing	33 830 (3.1)	2048 (3.4)	2547 (3.6)
Maternal smoking during pregnancy, cigarettes/d^b^			
0	828 128 (79.0)	46 487 (78.1)	55 825 (78.6)
1-9	107 991 (10.3)	6192 (10.4)	7478 (10.5)
≥10	58 154 (5.5)	3371 (5.7)	3633 (5.1)
Missing	54 565 (5.2)	3464 (5.8)	4053 (5.7)
Parity, No.^b^			
1	426 478 (40.6)	18 494 (31.0)	44 314 (62.4)
2	391 830 (37.4)	23 899 (40.2)	17 726 (25.0)
3	160 721 (15.3)	11 954 (20.1)	5967 (8.4)
≥4	69 809 (6.7)	5167 (8.7)	2982 (4.2)
Paternal age, y^b^			
<20	6193 (0.6)	172 (0.3)	308 (0.4)
20-29	399 219 (38.0)	15 822 (26.5)	24 919 (35.1)
30-39	534 640 (51.0)	33 675 (68.6)	36 502 (51.4)
≥40	102 160 (9.7)	9434 (15.9)	8636 (12.2)
Missing	6626 (0.6)	411 (0.7)	624 (0.9)
Maternal comorbidities and pregnancy complications^b^			
Hypertension	7028 (0.7)	699 (1.2)	942 (1.3)
Diabetes	9606 (0.9)	1741 (2.9)	1707 (2.4)
Maternal history of psychiatric disorders	22 349 (2.1)	2105 (3.5)	1917 (2.7)
Infections during pregnancy	4813 (0.5)	561 (0.9)	1604 (2.3)
Polyhydramnios	735 (0.1)	169 (0.3)	280 (0.4)
Oligohydramnios	7347 (0.7)	878 (1.5)	2303 (3.2)
Preeclampsia or eclampsia	24 265 (2.3)	2467 (4.1)	4604 (6.5)
Fetal malposition or malpresentation^b^			
Normal position or presentation	930 046 (88.7)	32 584 (55.8)	37 131 (52.3)
Breech presentation	7298 (0.7)	13 623 (22.9)	6876 (9.7)
Other malposition or malpresentation	34 414 (3.3)	2510 (4.2)	11 543 (16.3)
Missing	77 080 (7.3)	10 797 (18.1)	15 440 (21.7)
Paternal history of psychiatric disorders	19 585 (1.9)	1319 (2.2)	1475 (2.1)
**Neonatal characteristics**			
Male children^b^	533 140 (50.8)	30 138 (50.6)	39 191 (55.2)
Gestational age, wk^b^			
37	333 517 (32.0)	3439 (5.8)	16 522 (23.3)
38	46 371 (4.4)	8019 (11.5)	5234 (7.4)
39	120 847 (11.5)	32 894 (55.2)	9908 (14.0)
40	262 507 (25.0)	11 496 (19.3)	11 621 (16.4)
>40	285 596 (27.1)	3666 (6.2)	27 704 (39.0)
Small for gestational age^b^	17 913 (1.7)	1717 (2.9)	3363 (4.8)
Large for gestational age^b^	33 148 (3.2)	4806 (8.1)	4049 (5.7)
Indications for planned CD^b^			
Cephalopelvic disproportion	123 (0.01)	2808 (4.7)	NA
Indications for intrapartum CD^b^			
Cephalopelvic disproportion	661 (0.01)	NA	3636 (5.1)
Fetal distress, fetal hypoxia	58 509 (5.6)		27 063 (38.1)
Failed induction	36 985 (3.5)		8971 (12.6)
Placental disorders	8071 (0.8)		2394 (3.4)
Dystocia	117 294 (11.2)		23 457 (33.0)
Neurodevelopmental disorders in the children			
Attention-deficit/hyperactivity disorder^b^^,^^c^	45 692 (4.4)	2997 (5.1)	3568 (5.1)
Autism spectrum disorders^b^^,^^c^	16 432 (1.6)	1182 (2.0)	1416 (2.0)
Intellectual disability^b^^,^^c^	8783 (0.8)	693 (1.2)	856 (1.2)
Tic disorders^c^^,^^d^	2831 (0.3)	179 (0.3)	235 (0.3)
Communication disorders^b^^,^^c^	6234 (0.6)	482 (0.8)	514 (0.7)
Learning disorders^c^^,^^d^	5686 (0.5)	377 (0.6)	392 (0.6)
Psychiatric disorders in the children			
Anxiety and stress-related^b^^,^^e^	37 593 (3.7)	2034 (3.5)	2302 (3.3)
Depression and other mood^b^^,^^e^	29 205 (2.8)	1465 (2.5)	1804 (2.6)
Obsessive-compulsive^d^^,^^e^	5035 (0.5)	299 (0.5)	342 (0.5)
Eating^b^^,^^e^	8754 (0.9)	435 (0.8)	492 (0.7)
Bipolar^d^^,^^e^	3084 (0.3)	158 (0.3)	199 (0.3)
Schizophrenia and other psychotic^c^^,^^e^	1677 (0.2)	100 (0.2)	106 (0.2)

Abbreviations: CD, cesarean delivery; NA, not available.

^a^ Detailed description of each variable is presented in the eMethods 2 in the Supplement.

^b^ *P* < .001 determined with χ^2^ test.

^c^ Percentages were calculated in the respective cohort of 1 179 341 individuals born from 1990 to 2003.

^d^ *P* > .05 determined with χ^2^ test.

^e^ Percentages were calculated in the respective cohort of 740 352 individuals born from 1990 to 1997.

### Main Analyses

In the analysis adjusted for the children’s sex and birth year, planned and intrapartum CD were significantly associated with increased risk of any neurodevelopmental disorder (planned CD, HR, 1.20; 95% CI, 1.16-1.23; intrapartum CD, HR, 1.17; 95% CI, 1.13-1.20), ADHD (planned CD, HR, 1.17; 95% CI, 1.13-1.21; intrapartum CD, HR, 1.13; 95% CI, 1.09-1.17), ASD (planned CD, HR, 1.30; 95% CI, 1.22-1.37; intrapartum CD, HR, 1.26; 95% CI, 1.19-1.33), intellectual disability (planned CD, HR, 1.47; 95% CI, 1.36-1.58; intrapartum CD, HR, 1.48; 95% CI, 1.38-1.58) (Figure 2), any psychiatric disorder (planned CD, HR, 1.09; 95% CI, 1.05-1.14; intrapartum CD, HR, 1.09; 95% CI, 1.06-1.13), and anxiety disorders (planned CD, HR, 1.12; 95% CI, 1.06-1.17; intrapartum CD, HR, 1.07; 95% CI, 1.03-1.13) (Figure 3). Most of the associations attenuated but remained significant after adjusting for potential confounders: any neurodevelopmental disorder (planned CD, HR, 1.20; 95% CI, 1.16-1.24; intrapartum CD, HR, 1.21; 95% CI, 1.17-1.24), ADHD (planned CD, HR, 1.20; 95% CI, 1.15-1.25; intrapartum CD, HR, 1.19; 95% CI, 1.14-1.23), ASD (planned CD, HR, 1.27; 95% CI, 1.19-1.35; intrapartum CD, HR, 1.23; 95% CI, 1.16-1.30), intellectual disability (planned CD, HR, 1.40; 95% CI, 1.29-1.53; intrapartum CD, HR, 1.54; 95% CI, 1.43-1.66), any psychiatric disorder (planned CD, HR, 1.07; 95% CI, 1.03-1.12; intrapartum CD, HR, 1.10; 95% CI, 1.06-1.14), and anxiety disorders (planned CD, HR, 1.10; 95% CI, 1.05-1.16; intrapartum CD, HR, 1.08; 95% CI, 1.03-1.13) (Figure 2 and Figure 3). Furthermore, we adjusted for potential indications for CD and birth complications, observing a significant attenuation of the associations between planned and intrapartum CD and the outcomes (Figure 2 and Figure 3). Specifically, planned CD was robustly associated with increased risk of any neurodevelopmental disorder (HR, 1.17; 95% CI, 1.13-1.22), ADHD (HR, 1.17; 95% CI, 1.12-1.23), ASD (HR, 1.20; 95% CI, 1.10-1.31), intellectual disability (HR, 1.26; 95% CI, 1.14-1.39), communication disorders (HR, 1.14; 95% CI, 1.02-1.28), and learning disorders (HR, 1.15; 95% CI, 1.01-1.30) (Figure 2). Intrapartum CD was associated with increased risk of any neurodevelopmental disorder (HR, 1.10; 95% CI, 1.05-1.14), ADHD (HR, 1.10; 95% CI, 1.05-1.15), and intellectual disability (HR, 1.17; 95% CI, 1.06-1.28) (Figure 2). Intrapartum CD was no longer associated with ASD, communication disorders, any psychiatric disorder, anxiety disorders, and depressive disorders (Figure 2 and Figure 3). Planned or intrapartum CD were not associated with tic disorders, obsessive-compulsive disorder, bipolar disorder, or schizophrenia (Figure 2 and Figure 3).

### Sensitivity Analyses

The pattern of results for the associations between planned CD and the outcomes remained robust and was even stronger after applying restrictions (any neurodevelopmental disorder, HR, 1.26; 95% CI, 1.16-1.37; ADHD, HR, 1.32; 95% CI, 1.20-1.45; ASD, HR, 1.39; 95% CI, 1.19-1.61) (eTable 3 in the Supplement). However, the associations between intrapartum CD and the outcomes disappeared, with the exception of any neurodevelopmental disorder (HR, 1.21; 95% CI, 1.05-1.40). Results remained largely unchanged when assisted vaginal deliveries were excluded (eTable 4 in the Supplement).

### Post Hoc Analyses

To further explore the significant associations between CD and ADHD, ASD, and intellectual disability, we investigated potential effect modifications of measured covariates (eTable 5 in the Supplement). In the presence of maternal psychiatric history, the associations between planned CD and risk of ASD and ADHD in the children attenuated. Individuals delivered via planned or intrapartum CD with a malpresentation were more likely to be diagnosed with ASD, ADHD, and intellectual disability. Failed induction largely explained the increased risk of ASD, ADHD, and intellectual disability when delivered by intrapartum CD. Individuals presenting with fetal distress at birth and delivered via intrapartum CD were at higher risk of being diagnosed with intellectual disability later in life. When stratifying by gestational age while adjusting for all potential confounders, planned and intrapartum CD were consistently associated with an increased risk of any neurodevelopmental disorder (at 39 weeks, planned CD: HR, 1.22; 95% CI, 1.12-1.33; intrapartum CD: HR, 1.26; 95% CI, 1.16-1.36), ADHD (planned CD: HR, 1.17; 95% CI, 1.06-1.30; intrapartum CD: HR, 1.25; 95% CI, 1.13-1.38), ASD (planned CD: HR, 1.30; 95% CI, 1.10-1.52; intrapartum CD: HR, 1.33; 95% CI, 1.13-1.55), and intellectual disability (planned CD: HR, 1.43; 95% CI, 1.15-1.78; intrapartum CD: HR, 1.67; 95% CI, 1.37-2.04), whereas the associations between CD and ASD largely attenuated and became nonsignificant except at 39 weeks (eTable 6 in the Supplement).

We repeated the main analyses separately for individuals with ASD with and without intellectual disability, because these conditions often co-occur and may mask true associations with one or the other condition. We found that risks of ASD without intellectual disability remained statistically significant (fully adjusted, planned CD, HR, 1.21; 95% CI, 1.12-1.30; intrapartum CD, HR, 1.15; 95% CI, 1.07-1.22), whereas no association between CD and ASD with intellectual disability was observed (eTable 7 in the Supplement).

### Within-Family Analyses

We performed within-family analyses for the associations between CD and any neurodevelopmental disorder, ADHD, ASD, and intellectual disability (Table 2), because these associations remained significant in the main analyses after adjusting for all measured confounders. In the full-cousin, paternal and maternal half-sibling, and full-sibling comparison, the risk only remained in paternal half siblings for any neurodevelopmental disorder (HR, 1.33; 95% CI, 1.03-1.73), ADHD (HR, 1.40; 95% CI, 1.04-1.89), ASD (HR, 1.98; 95% CI, 1.08-3.63), and intellectual disability (HR, 2.14; 95% CI, 1.15-3.99) in those born via planned CD. In the full-cousin and maternal half- and full-sibling comparisons, the associations between CD and the outcomes attenuated to the null (ie, became nonsignificant) except in the maternal half-sibling comparisons, where we observed a reduced risk of ASD for planned CD (HR, 0.30; 95% CI, 0.10-0.94). In our cohort, we found that mothers who delivered via CD were less likely to have more than 1 child than mothers who delivered vaginally (66% vs 51% vs 56%, *P* < .001, if the mother delivered the first child via vaginal delivery, planned CD, and intrapartum CD, respectively).

**Table 2.** Estimated Risk of Neurodevelopmental Disorders in Relatives of Individuals Delivered by Planned and Intrapartum Cesarean Delivery Compared With Individuals Delivered by Vaginal Delivery

Cesarean delivery	HR (95% CI)			
	Full maternal cousins^a^	Half siblings		Full siblings^d^
		Paternal^b^	Maternal^c^	
**Any neurodevelopmental disorder**^**e**^				
Planned	1.06 (0.90-1.25)	1.33 (1.03-1.73)^f^	0.96 (0.61-1.51)	0.93 (0.81-1.06)
Intrapartum	1.11 (0.97-1.26)	0.96 (0.78-1.19)	0.99 (0.76-1.30)	1.07 (0.96-1.21)
**Attention-deficit/hyperactivity disorder**				
Planned	1.07 (0.87-1.32)	1.40 (1.04-1.89)^f^	1.12 (0.68-1.84)	0.92 (0.81-1.06)
Intrapartum	1.09 (0.93-1.28)	1.01 (0.79-1.30)	1.11 (0.82-1.52)	1.07 (0.95-1.20)
**Autism spectrum disorders**				
Planned	1.07 (0.79-1.45)	1.98 (1.08-3.63)^f^	0.30 (0.10-0.94)^f^	0.91 (0.79-1.04)
Intrapartum	1.16 (0.89-1.51)	1.24 (0.78-1.97)	1.18 (0.65-2.14)	1.07 (0.95-1.20)
**Intellectual disability**				
Planned	1.19 (0.83-1.72)	2.14 (1.15-3.99)^f^	1.21 (0.42-3.48)	0.88 (0.72-1.09)
Intrapartum	1.31 (0.94-1.82)	1.28 (0.75-2.19)	0.78 (0.36-1.71)	1.08 (0.91-1.28)

Abbreviation: HR, hazard ratio.

^a^ This model was adjusted for child’s sex and year of birth, gestational age, age of mother and father at birth, parity, mother’s highest education level at birth, maternal and paternal history of psychiatric disorders, maternal diabetes, maternal infections during pregnancy, and large for gestational age. For intrapartum CD, we further adjusted for dystocia, failed induction, and fetal distress.

^b^ This model was adjusted for the previously named covariates except for paternal history of psychiatric disorders.

^c^ This model was adjusted for the previously named covariates except for maternal history of psychiatric disorders and further adjusted for birth order.

^d^ This model was adjusted for the previously named covariates except for maternal and paternal history of psychiatric disorders and further adjusted for birth order.

^e^ Any neurodevelopmental disorders included any diagnosis of attention-deficit/hyperactivity disorder, autism spectrum disorders, intellectual disability, tic disorders, communication disorders, or learning disorders.

^f^ Indicates significant estimates. In the within-family analyses, we identified full siblings (ie, share the same biologic mother and father), maternal half siblings (ie, share the same biologic mother but different fathers), paternal half siblings (ie, share the same biologic father but different mothers), and maternal full cousins (ie, mothers are full siblings).

## Discussion

In this study including nearly 1.2 million deliveries, we found that children born via planned or intrapartum CD had a 10% to 30% increased risk of being diagnosed with any neurodevelopmental disorder, ADHD, and intellectual disability, compared with children born via vaginal delivery. We also observed an increased risk of ASD, communication disorders, and learning disorders among children born via planned CD. However, the observed associations attenuated to the null after adjusting for familial factors. Overall, the results do not suggest a causal association between CD and neurodevelopmental and psychiatric disorders.

Similar to our results, previous meta-analyses found that CD was associated with a 10% to 30% increased risk of ASD and ADHD.^9,10^ Other studies have found comparable risks for planned and intrapartum CD (in some studies, the 2 types of CD were referred to as elective and emergency CD, respectively),^9,10^ whereas we found the estimates to be higher for planned CD. In our study, the associations between intrapartum CD and the outcomes greatly attenuated after adjusting for fetal distress, dystocia, and failed induction. These findings were further confirmed in the subgroup analyses, where the association between intrapartum CD and the outcomes was completely attenuated in the group with failed induction (a proxy for maternal risk factors that may complicate the birth). Although hypoxia may affect brain development and result in neurodevelopmental impairments,^34,35^ our study was unable to determine whether these disorders were caused by the hypoxia-related factors.

Familial factors were suggested to explain the observed associations between CD and neurodevelopmental outcomes.^16,17,18,19^ To explore the origin of familial liability, we enriched our quasi-experimental family design by adding half-sibling and cousin comparisons, which by design could control for shared factors in the extended families.^36,37,38^ If the familial liability were due to the shared environment, the magnitude of the associations should differ between paternal and maternal half siblings, because they share equivalent genetic factors, but maternal half siblings tend to share more family environment than paternal half siblings, because children usually reside with mothers if the parents are separated.^39^ We observed attenuated but significant associations between planned CD and any neurodevelopmental disorder, ADHD, ASD, and intellectual disability in paternal half siblings, but no association between CD and any outcome in maternal half-sibling comparisons, indicating the potential role of maternal factors in these associations. Furthermore, we did not find higher risks in full siblings than in maternal half siblings, suggesting a minor role of the children’s shared genetics, because these relatives share similar family environment but full siblings share a higher proportion of genetics than half siblings. Overall, the family results suggest that the associations between CD and neurodevelopmental disorders were likely explained by familial factors, especially shared maternal factors.

To our knowledge, this is the first study to control for a comprehensive list of CD indications when examining the associations between CD and neurodevelopmental and psychiatric outcomes in the children. By accounting for pregnancy and birth complications that may indicate or increase the risk of ending a delivery with CD, we were able to address confounding by indication. Adjusting for CD indications largely attenuated the associations between intrapartum CD, but not planned CD, and the outcomes. A further exploration of the association between planned CD and the outcomes is warranted.

### Strengths and Limitations

Our study has several strengths. The nearly 1.2 million births provided sufficient statistical power to study less common outcomes that have not been studied previously (eg, communication disorders and learning disorders). The risk of selection bias was low owing to the use of register linkage data. Furthermore, we carefully designed the within-family analyses considering that the exposure status in discordant pairs may not be proportionally distributed in maternal half and full siblings. That is, firstborn children were more likely to be unexposed (being delivered vaginally), whereas second-born children were more likely to be exposed (being delivered through CD), rather than vice versa. Moreover, mothers delivering vaginally after a CD may be healthier (ie, at lower risk of developing birth complications and therefore less likely to deliver via CD) than mothers delivering via CD after a CD or mothers who do not have more children after a CD. We adjusted for birth order to address this bias.

This study also has some limitations. Our sample size did not allow precise estimations for some rare outcomes or outcomes with later onset, particularly for some psychiatric disorders, although a trimmed cohort was used for these outcomes to allow for a longer follow-up time. The outcomes might be underreported because many individuals do not seek help, coverage of the National Patient Register is incomplete between 1997 and 2001, and the National Patient Register does not record diagnoses from general practitioners or nonspecialists.^27^ However, this would not overestimate the results, because the underreporting is unlikely to differ by exposure status. Second, the registers do not include information on other perinatal factors thought to have an effect on child microbiota development (eg, exposure to antibiotics during pregnancy and breastfeeding),^40,41,42,43^ although the evidence of these effects is inconclusive.^16,19,44,45,46,47,48,49^ Third, generalizability may be a concern, because the CD rate varies greatly between countries.^50^ Our findings may only be generalizable to countries with similar settings where most CDs are performed in the presence of medical indications. Furthermore, limitations inherent to family designs should be considered. Within-family designs can help explore familial liability and inferring, but they cannot prove causality. They also provide less precise estimates owing to reduced statistical power and are more susceptible to nonshared confounding.^51^ Moreover, both the studied exposures and outcomes may lead to a reproductive stoppage^52,53,54,55,56^ in the family that may potentially threaten the generalizability of sibling results. In our cohort, mothers who delivered via CD were more likely to curtail their reproduction than mothers who delivered vaginally. Therefore, we performed analyses in paternal half siblings and cousins who do not share the same mothers and thus are not affected by the firstborns’ delivery mode.

## Conclusions

The results of this cohort study suggest that birth via planned or intrapartum CD was associated with a 10% to 30% increased risk of neurodevelopmental disorders in the children, such as ADHD and intellectual disability, compared with births via vaginal delivery. However, our study also suggested that these associations may be largely explained by familial factors. Those conducting observational studies on the association between prenatal risk factors and child outcomes should be wary of causal claims, because the association may be spurious owing to unmeasured confounders.

## Notes

Supplement. **eMethods 1.** Description of Swedish Registers Used in the Study **eMethods 2.** Details Regarding the Measured Covariates Included in the Adjustment Models **eTable 1.** Diagnostic Codes and Minimum Age for Each Outcome Variable **eTable 2.** Mean, Median, and Range of the Age at First Recorded Diagnosis for Each Neurodevelopmental and Psychiatric Disorder **eTable 3.** Estimated Risk of Neurodevelopmental Disorders Among Individuals Delivered by Planned and Intrapartum Cesarean Delivery Compared to Vaginally-Delivered Individuals, in a Restricted Population **eTable 4.** Estimated Risk of Neurodevelopmental and Psychiatric Disorders Among Individuals Delivered by Planned and Intrapartum Cesarean Delivery Compared to Individuals Born by Unassisted Vaginal Delivery **eTable 5.** Subgroup Analyses for the Estimated Risk of Autism Spectrum Disorders, Attention-Deficit/Hyperactivity Disorder, and Intellectual Disability in Individuals Delivered by Planned and Intrapartum Cesarean Delivery Compared to Vaginally-Delivered Individuals, Stratified by a Number of Covariates and Testing Interactions for Significance **eTable 6.** Estimated Risk of Neurodevelopmental Disorders Among Individuals Delivered by Planned and Intrapartum Cesarean Delivery Compared to Vaginally-Delivered Individuals, by Gestational Age **eTable 7.** Estimated Risk of Autism Spectrum Disorder With and Without Intellectual Disability Among Individuals Delivered by Planned and Intrapartum Cesarean Delivery, Compared to Vaginally-Delivered Individuals **eReferences**
