# Supplementary material for: Assessment of Cesarean Delivery and Neurodevelopmental and Psychiatric Disorders in the Children of a Population-Based Swedish Birth Cohort
Source: JAMA Netw Open. 2021 Mar 5;4(3):e210837. doi: 10.1001/jamanetworkopen.2021.0837 (PMC7936261; doi:10.1001/jamanetworkopen.2021.0837)
Supplement: Supplement. — eMethods 1. Description of Swedish Registers Used in the Study eMethods 2. Details Regarding the Measured Covariates Included in the Adjustment Models eTable 1. Diagnostic Codes and Minimum Age for Each Outcome Variable eTable 2. Mean, Median, and Range of the Age at First Recorded Diagnosis for Each Neurodevelopmental and Psychiatric Disorder eTable 3. Estimated Risk of Neurodevelopmental Disorders Among Individuals Delivered by Planned and Intrapartum Cesarean Delivery Compared to Vaginally-Delivered Individuals, in a Restricted Population eTable 4. Estimated Risk of Neurodevelopmental and Psychiatric Disorders Among Individuals Delivered by Planned and Intrapartum Cesarean Delivery Compared to Individuals Born by Unassisted Vaginal Delivery eTable 5. Subgroup Analyses for the Estimated Risk of Autism Spectrum Disorders, Attention-Deficit/Hyperactivity Disorder, and Intellectual Disability in Individuals Delivered by Planned and Intrapartum Cesarean Delivery Compared to Vaginally-Delivered Individuals, Stratified by a Number of Covariates and Testing Interactions for Significance eTable 6. Estimated Risk of Neurodevelopmental Disorders Among Individuals Delivered by Planned and Intrapartum Cesarean Delivery Compared to Vaginally-Delivered Individuals, by Gestational Age eTable 7. Estimated Risk of Autism Spectrum Disorder With and Without Intellectual Disability Among Individuals Delivered by Planned and Intrapartum Cesarean Delivery, Compared to Vaginally-Delivered Individuals eReferences [file jamanetwopen-e210837-s001.pdf]

Supplementary Online Content

Zhang T, Brander G, Mantel Ä, et al. Assessment of cesarean delivery and neurodevelopmental and psychiatric disorders in the children of a population-based Swedish birth cohort. *JAMA Netw Open*. 2021;4(3):e210837. doi:10.1001/jamanetworkopen.2021.0837

eMethods 1. Description of Swedish Registers Used in the Study

eMethods 2. Details Regarding the Measured Covariates Included in the Adjustment Models

eTable 1. Diagnostic Codes and Minimum Age for Each Outcome Variable

eTable 2. Mean, Median, and Range of the Age at First Recorded Diagnosis for Each Neurodevelopmental and Psychiatric Disorder

eTable 3. Estimated Risk of Neurodevelopmental Disorders Among Individuals Delivered by Planned and Intrapartum Cesarean Delivery Compared to Vaginally-Delivered Individuals, in a Restricted Population

eTable 4. Estimated Risk of Neurodevelopmental and Psychiatric Disorders Among Individuals Delivered by Planned and Intrapartum Cesarean Delivery Compared to Individuals Born by Unassisted Vaginal Delivery

eTable 5. Subgroup Analyses for the Estimated Risk of Autism Spectrum Disorders, Attention-Deficit/Hyperactivity Disorder, and Intellectual Disability in Individuals Delivered by Planned and Intrapartum Cesarean Delivery Compared to Vaginally-Delivered Individuals, Stratified by a Number of Covariates and Testing Interactions for Significance

eTable 6. Estimated Risk of Neurodevelopmental Disorders Among Individuals Delivered by Planned and Intrapartum Cesarean Delivery Compared to Vaginally-Delivered Individuals, by Gestational Age

eTable 7. Estimated Risk of Autism Spectrum Disorder With and Without Intellectual Disability Among Individuals Delivered by Planned and Intrapartum Cesarean Delivery, Compared to Vaginally-Delivered Individuals

eReferences

This supplementary material has been provided by the authors to give readers additional information about their work.

© 2021 Zhang T et al. *JAMA Network Open.*

eMethods 1. . Description of Swedish Registers Used in the Study

(1) The **Medical Birth Register** includes data on more than 99% of all pregnancies and deliveries in Sweden since 1973. The register is constructed based on information from medical birth reports, which are summarized medical records on a standard form prepared by secretaries at obstetric clinics. From 1982, an updated form is used, concentrating on antenatal care of the mother, the delivery record, and the record for the pediatric examination of the newborns. At the same time, the contents of the register were expanded and, for some categories, check boxes instead of medical codes were introduced.

(2) The **Multi-Generation Register** connects every person born in Sweden since 1932, and ever registered as living in the country from 1961, with their biological or adoptive parents. This allows for identifying relatives of different genetic relatedness, such as siblings. The register contains information on 100% of mothers and 98% of fathers of individuals born after 1961.

(3) The **National Patient Register** covers all primary medical diagnoses and up to eight additional diagnoses given from inpatient hospital admissions since 1969, with diagnoses based on the International Classification of Diseases (ICD), eighth (ICD-8; 1969–1986), ninth (ICD-9; 1987–1996), and tenth (ICD-10; 1997–2013) revisions. All counties in Sweden started recording specialist psychiatric care from 1973 and, from 2001, outpatient specialist care is also included.

(4) The **Prescribed Drug Register** contains data on all dispensed prescriptions of medication to the whole population of Sweden since July 1, 2005. The information includes type of medication classified according to the Anatomical Therapeutic Chemical classification system, date of the prescription, and dosage.

(5) The **Longitudinal Integration Database for Health Insurance and Labour Studies** covers the adult Swedish population aged older than 16 years registered on December 31 each year since 1990 (and since 2010 for individuals older than 15 years) with information on education, income, and occupation.

(6) The **Total Population Register** contains information about emigration since 1961 and immigration since 1969 from and to Sweden.

(7) The **Cause of Death Register** includes dates and causes of more than 99% of all deaths of Swedish residents, both in Sweden and abroad, since 1961.

© 2021 Zhang T et al. *JAMA Network Open.*

eMethods 2. Details Regarding the Measured Covariates Included in the Adjustment Models

Information about all potential, measured confounders was collected from the Medical Birth Register, unless otherwise specified. Offspring's year of birth and sex were included in all models. Data on maternal age at delivery, parity, fetus small/large for gestational age, and gestational age was obtained. Small or large for gestational age were defined as a birth weight of more than two standard deviations below or above the mean weight for gestational age, respectively, according to the Scandinavian fetal growth curve adjusted for sex.[1] Data on paternal age at delivery was also retrieved. Data on mother's highest education in years was obtained from the Longitudinal Integration Database for Health Insurance and Labour Studies. We also adjusted for the following parental, perinatal, and neonatal covariates: (a) *maternal and paternal history of psychiatric disorders* was identified as having any psychiatric disorder diagnosis (i.e., including any of the following codes: 290-319 in ICD-9 or F00-F99 in ICD-10) recorded in the National Patient Register prior to delivery; (b) *maternal smoking during pregnancy* was recorded at the first antenatal visit (within the first 14 gestational weeks for 90% of pregnant women) and was categorized as no daily smoking, 1-9 cigarettes per day, and more than 10 cigarettes per day; (c) *maternal hypertensive disorders* included pre-existing hypertension and gestational hypertension (642 in ICD-9; O10, O11, O13 and O16 in ICD-10, respectively); (d) *maternal diabetes* included pre-existing diabetes mellitus and gestational diabetes mellitus (648A and 648W in ICD-9, respectively; O24 in ICD-10); (e) *maternal infections during pregnancy*, including genitourinary tract (O23 in ICD-10) and chorioamnionitis (658E and 762H in ICD-9; O41.1 in ICD-10); (f) *polyhydramnios* was identified with the code 657 in ICD-9 and O40 in ICD-10; (g) *oligohydramnios* was identified with the code 658A in ICD-9 and O41.0 in ICD-10; (h) *pre-eclampsia* and *eclampsia* were identified with the codes 642E, F, G, H, X in ICD-9 and O14-15 in ICD-10; (i) *labor position/presentation* was recorded in the Medical Birth Register and divided into three categories: normal position/presentation, breech presentation, and other malposition/malpresentation (e.g., face or brow presentation, persistent occiput posterior position); (j) *pelvic disproportion* that indicates a planned cesarean delivery was identified with the code 653X in ICD-9 and O33.9 in ICD-10; (k) *pelvic disproportion* that indicates an intrapartum cesarean delivery was identified with the code 653E in ICD-9 and O65 in ICD-10; (l) *fetal distress, hypoxia or asphyxia* were identified with the codes 656D and 768 in ICD-9 and O36.3, O68, and P21 in ICD-10; (m) *failed induction* of labor was identified with the codes 659A and 659B in ICD-9 and O61 and O75.5 in ICD-10, including failed medical and instrumental inductions; (n) *placental disorders* included placenta previa, placenta abruptio, and unspecific bleeding and were identified with the codes 641 in ICD-9 and O44-46 in ICD-10; (o) *dystocia* was identified with the codes 660X and 661 in ICD-9 and O62 in ICD-10.

eTable 1. Diagnostic Codes and Minimum Age for Each Outcome Variable

Disorder / group of disorders	Codes[a]	Minimum age for diagnosis
**Neurodevelopmental disorders**		
Attention-deficit/hyperactivity disorder	ICD-9 314<br>ICD-10 F90<br>Medication for ADHD:[b] amphetamine [ATC code N06BA01], dexamphetamine [ATC code N06BA02], methylphenidate [ATC code N06BA04], atomoxetine [ATC code N06BA09], and lisdexamphetamine [ATC code N06BA12]	3
Autism spectrum disorders	ICD-9 299<br>ICD-10 F84.0, .1, .3, .5, .8, .9	1
Intellectual disability	ICD-9 317-319<br>ICD-10 F70, F71, F72, F73, F78, F79	1
Tic disorders (including Tourette syndrome and chronic tic disorders)[c]	ICD-9 307C<br>ICD-10 F95.0, .1, .2, .8, .9	3
Communication disorders	ICD-9 315D<br>ICD-10 F80	3
Learning disorders	ICD-9 315A, 315B<br>ICD-10 F81, R48	3
**Psychiatric disorders**		
Anxiety and stress-related disorders	ICD-9 300A, 300C, 308, 309<br>ICD-10 F40, F41, F43	6
Depression and other mood disorders	ICD-9 296B, 296X, 298A, 300E, 311<br>ICD-10 F32, F33, F34 (excluding F34.0), F38, F39	6
Obsessive-compulsive disorder	ICD-9 300D<br>ICD-10 F42	6
Eating disorders	ICD-9 307B, 307F<br>ICD-10 F50.0-F50.3, F50.9	8
Bipolar disorders	ICD-9 296A, 296C, 296D, 296E, 296W, 296X<br>ICD-10 F25.0, F30, F31, F34.0	10
Schizophrenia and other psychotic disorders	ICD-9 295 (excluding 295F), 297, 298 (excluding 298A)<br>ICD-10 F20, F21, F22, F23, F24, F25 (excluding F25.0), F28, F29	10

Abbreviations: ATC: Anatomical Therapeutic Chemical; ICD: International Classification of Diseases
[a] According to Swedish ICD codes.
[b] It was possible to identify all dispensed medications approved in Sweden for the management of ADHD from the PDR. The listed medications were approved in Sweden for the management of ADHD during the time period relevant for the study.
[c] Tourette syndrome and chronic tic disorder were identified using a previously validated algoritm.[2]

© 2021 Zhang T et al. *JAMA Network Open.*

eTable 2. Mean, Median, and Range of the Age at First Recorded Diagnosis for Each Neurodevelopmental and Psychiatric Disorder

	Median age	Mean age (SD)	Range
**Neurodevelopmental disorders**			
Autism spectrum disorders	13.1	12.9 (5.1)	1.1 – 23.9
Intellectual disability	10.8	10.9 (5.1)	1.0 – 23.9
Attention-deficit/hyperactivity disorder	13.0	13.1 (4.2)	3.0 – 23.9
Tic disorders	11.4	11.8 (3.7)	3.2 – 23.8
Communication disorders	6.2	7.6 (3.6)	3.0 – 22.2
Learning disorders	13.1	13.3 (3.4)	3.1 – 23.5
**Psychiatric disorders**			
Anxiety and stress-related disorders	17.4	17.1 (3.4)	6.0 – 23.9
Depression and other mood disorders	17.3	17.3 (2.8)	6.2 – 24.0
Obsessive-compulsive disorder	16.0	15.8 (3.7)	6.0 – 23.8
Eating disorder	16.1	16.3 (2.7)	8.0 – 23.9
Bipolar disorders	18.5	18.3 (2.8)	10.0 – 23.9
Schizophrenia and other psychotic disorders	18.9	18.6 (2.6)	10.2 – 23.9

Abbreviations: SD: standard deviation.

© 2021 Zhang T et al. *JAMA Network Open.*

eTable 3. Estimated Risk of Neurodevelopmental Disorders Among Individuals Delivered by Planned and Intrapartum Cesarean Delivery Compared to Vaginally-Delivered Individuals, in a Restricted Population

	Adjusted,[a] HR (95% CIs)
**Any neurodevelopmental disorder[b]**	
Planned CD	**1.26 (1.16, 1.37)**
Intrapartum CD	**1.21 (1.05, 1.40)**
**Attention-deficit/hyperactivity disorder**	
Planned CD	**1.32 (1.20, 1.45)**
Intrapartum CD	1.15 (0.96, 1.37)
**Autism spectrum disorders**	
Planned CD	**1.39 (1.19, 1.61)**
Intrapartum CD	1.30 (0.99, 1.70)
**Intellectual disability**	
Planned CD	1.23 (0.98, 1.53)
Intrapartum CD	0.96 (0.62, 1.50)

Abbreviations: CIs: confidence intervals; CD: cesarean delivery; HR: hazard ratio.

Note: We applied restrictions to yield a more homogenous population that had the characteristics of the following: mothers who were aged between 20 to 35 years old; parity less than three; no reported smoking behaviors during pregnancy; no recorded comorbidities (e.g., psychiatric disorders [both parents], hypertensive disorders, diabetes, and infections during pregnancy); no recorded pregnancy complications (e.g., pelvic disproportion, placenta disorders, polyhydramnios, oligohydramnios, pre-eclampsia/eclampsia); no birth complications (e.g., dystocia, failed induction, fetal distress); and fetuses were in normal position/presentation with normal weight for gestational age.

[a] Model adjusted for offspring's sex and year of birth, gestational age, age of mother and father at birth, mother's highest education level at birth, and parity.
[b] Any neurodevelopmental disorder includes any of the following diagnoses: attention-deficit/hyperactivity disorder, autism spectrum disorders, intellectual disability, tic disorders, communication disorders, and learning disorders.

© 2021 Zhang T et al. *JAMA Network Open.*

eTable 4. Estimated Risk of Neurodevelopmental and Psychiatric Disorders Among Individuals Delivered by Planned and Intrapartum Cesarean Delivery Compared to Individuals Born by Unassisted Vaginal Delivery

	Adjusted for sex and year of birth, HR (95% CIs)
**Any neurodevelopmental disorder[a]**	
Planned CD	**1.20 (1.16, 1.23)**
Intrapartum CD	**1.16 (1.13, 1.20)**
**Attention-deficit/hyperactivity disorder**	
Planned CD	**1.17 (1.12, 1.21)**
Intrapartum CD	**1.13 (1.09, 1.16)**
**Autism spectrum disorder**	
Planned CD	**1.30 (1.23, 1.38)**
Intrapartum CD	**1.26 (1.20, 1.34)**
**Intellectual disability**	
Planned CD	**1.47 (1.36, 1.59)**
Intrapartum CD	**1.48 (1.38, 1.59)**
**Tic disorders**	
Planned CD	1.13 (0.97, 1.31)
Intrapartum CD	**1.17 (1.02, 1.34)**
**Communication disorders**	
Planned CD	**1.22 (1.11, 1.34)**
Intrapartum CD	1.09 (0.99, 1.19)
**Learning disorders**	
Planned CD	**1.20 (1.08, 1.33)**
Intrapartum CD	1.00 (0.91, 1.11)
**Any psychiatric disorder[b]**	
Planned CD	**1.09 (1.05, 1.14)**
Intrapartum CD	**1.10 (1.06, 1.13)**
**Anxiety and stress-related disorders**	
Planned CD	**1.12 (1.06, 1.17)**
Intrapartum CD	**1.07 (1.03, 1.12)**
**Depression and other mood disorders**	
Planned CD	**1.06 (1.01, 1.12)**
Intrapartum CD	**1.11 (1.06, 1.17)**
**Eating disorder**	
Planned CD	1.01 (0.91, 1.12)
Intrapartum CD	1.01 (0.92, 1.13)
**Obsessive-compulsive disorder**	
Planned CD	**1.13 (0.98, 1.30)**
Intrapartum CD	**1.18 (1.04, 1.33)**
**Bipolar disorders**	
Planned CD	1.11 (0.94, 1.31)
Intrapartum CD	**1.17 (1.01, 1.36)**
**Schizophrenia and other psychotic disorders**	
Planned CD	**1.35 (1.09, 1.65)**
Intrapartum CD	1.12 (0.91, 1.37)

Abbreviations: CIs: confidence intervals; CD: cesarean delivery; HR: hazard ratio.

[a] Any neurodevelopmental disorder includes any of the following diagnoses: attention-deficit/hyperactivity disorder, autism spectrum disorders, intellectual disability, tic disorders, communication disorders, and learning disorders.
[b] Any psychiatric disorder includes any of the following diagnoses: anxiety and stress-related disorders, obsessive-compulsive disorder, depression and other mood disorders, eating disorders, bipolar disorders, and schizophrenia and other psychotic disorder.

© 2021 Zhang T et al. *JAMA Network Open.*

eTable 5. Subgroup Analyses for the Estimated Risk of Autism Spectrum Disorders, Attention-Deficit/Hyperactivity Disorder, and Intellectual Disability in Individuals Delivered by Planned and Intrapartum Cesarean Delivery Compared to Vaginally-Delivered Individuals, Stratified by a Number of Covariates and Testing Interactions for Significance

(i) Maternal and paternal comorbidities and pregnancy complications

	Maternal hypertensive disorders			Maternal diabetes			Maternal history of psychiatric disorders		
	Yes	No	p-value	Yes	No	p-value	Yes	No	p-value
**ASD**									
Planned CD	1.35 (0.81, 2.24)	1.43 (1.35, 1.52)	0.83	1.37 (1.00, 1.88)	1.42 (1.34, 1.51)	0.81	1.30 (1.04, 1.62)	1.41 (1.33, 1.50)	0.52
Intrapartum CD	1.63 (1.06, 2.50)	1.39 (1.32, 1.47)	0.49	1.44 (1.06, 1.97)	1.39 (1.32, 1.47)	0.85	1.00 (0.77, 1.29)	1.41 (1.34, 1.50)	**0.01**
**ADHD**									
Planned CD	1.28 (0.91, 1.80)	1.30 (1.26, 1.35)	0.91	1.27 (1.03, 1.56)	1.30 (1.25, 1.35)	0.86	1.25 (1.09, 1.42)	1.28 (1.23, 1.33)	0.88
Intrapartum CD	1.45 (1.08, 1.95)	1.27 (1.22, 1.31)	0.31	1.46 (1.18, 1.77)	1.26 (1.22, 1.30)	0.17	0.99 (0.84, 1.15)	1.28 (1.23, 1.33)	**0.002**
**Intellectual disability**									
Planned CD	2.27 (1.30, 3.98)	1.50 (1.39, 1.62)	0.25	1.24 (0.82, 1.86)	1.50 (1.39, 1.63)	0.34	1.84 (1.39, 2.43)	1.46 (1.35, 1.59)	0.15
Intrapartum CD	1.43 (0.77, 2.64)	1.54 (1.43, 1.65)	0.83	0.94 (0.59, 1.48)	1.55 (1.44, 1.66)	**0.03**	1.26 (0.90, 1.77)	1.54 (1.44, 1.65)	0.23

Cont. eTable 5 (i)

	Paternal history of psychiatric disorders			Infections during pregnancy			Polyhydramnios		
	Yes	No	p-value	Yes	No	p-value	Yes	No	p-value
**ASD**									
Planned CD	1.29 (0.96, 1.74)	1.43 (1.35, 1.52)	0.51	0.58 (0.27, 1.24)	1.44 (1.36, 1.53)	**0.03**	0.87 (0.30, 2.51)	1.43 (1.35, 1.52)	0.36
Intrapartum CD	1.21 (0.91, 1.61)	1.41 (1.33, 1.49)	0.31	0.60 (0.37, 0.98)	1.42 (1.34, 1.50)	**<0.001**	1.23 (0.57, 2.70)	1.40 (1.32, 1.48)	0.78
**ADHD**									
Planned CD	1.31 (1.12, 1.54)	1.30 (1.25, 1.35)	0.81	0.81 (0.54, 1.22)	1.31 (1.26, 1.36)	**0.03**	1.00 (0.52, 1.92)	1.30 (1.26, 1.35)	0.42
Intrapartum CD	1.17 (0.99, 1.37)	1.27 (1.23, 1.32)	0.33	1.02 (0.80, 1.30)	1.27 (1.23, 1.32)	0.08	0.97 (0.56, 1.68)	1.27 (1.23, 1.31)	0.34
**Intellectual disability**									
Planned CD	1.77 (1.26, 2.48)	1.49 (1.38, 1.62)	0.38	0.76 (0.31, 1.91)	1.52 (1.41, 1.64)	0.14	1.22 (0.50, 3.05)	1.54 (1.43, 1.65)	0.63
Intrapartum CD	1.20 (0.82, 1.76)	1.55 (1.44, 1.66)	0.19	0.88 (0.51, 1.54)	1.55 (1.44, 1.66)	**0.05**	0.52 (0.18, 1.51)	1.57 (1.47, 1.67)	**0.04**

© 2021 Zhang T et al. *JAMA Network Open.*

Cont. eTable 5 (i)

	Oligohydramnios			Pre-eclampsia/eclampsia			Fetal presentation		
	Yes	No	p-value	Yes	No	p-value	Normal	Breech or other malpresentation	p-value
**ASD**									
Planned CD	1.43 (0.92, 2.23)	1.42 (1.34, 1.51)	0.99	1.12 (0.85, 1.49)	1.44 (1.35, 1.53)	0.09	1.48 (1.37, 1.60)	1.80 (1.62, 1.99)	**0.01**
Intrapartum CD	1.45 (1.06, 1.98)	1.38 (1.31, 1.46)	0.76	1.21 (0.98, 1.50)	1.40 (1.32, 1.48)	0.21	1.39 (1.29, 1.49)	1.88 (1.71, 1.06)	**<0.001**
**ADHD**									
Planned CD	1.07 (0.80, 1.43)	1.30 (1.25, 1.35)	0.17	1.18 (0.99, 1.41)	1.31 (1.26, 1.36)	0.29	1.32 (1.26, 1.39)	1.72 (1.62, 1.83)	**<0.001**
Intrapartum CD	0.96 (0.78, 1.18)	1.27 (1.22, 1.31)	**0.01**	1.12 (0.97, 1.29)	1.27 (1.23, 1.32)	0.10	1.28 (1.22, 1.34)	1.71 (1.61, 1.81)	**<0.001**
**Intellectual disability**									
Planned CD	1.21 (0.67, 2.16)	1.51 (1.40, 1.63)	0.43	1.24 (0.86, 1.78)	1.52 (1.40, 1.64)	0.27	1.54 (1.39, 1.71)	1.59 (1.40, 1.81)	**0.02**
Intrapartum CD	1.06 (0.70, 1.62)	1.54 (1.43, 1.65)	0.09	1.24 (0.93, 1.64)	1.55 (1.44, 1.66)	0.13	1.54 (1.40, 1.69)	1.66 (1.48, 1.87)	**<0.001**

(ii) Neonatal characteristics

	Small for gestational age			Large for gestational age		
	Yes	No	p-value	Yes	No	p-value
**ASD**						
Planned CD	1.47 (1.11, 1.94)	1.42 (1.33, 1.50)	0.77	1.54 (1.26, 1.88)	1.40 (1.32, 1.49)	0.36
Intrapartum CD	1.50 (1.22, 1.86)	1.37 (1.29, 1.45)	0.35	1.31 (1.04, 1.64)	1.40 (1.32, 1.48)	0.60
**ADHD**						
Planned CD	1.51 (1.27, 1.80)	1.29 (1.24, 1.34)	0.07	1.48 (1.31, 1.68)	1.28 (1.23, 1.33)	**0.03**
Intrapartum CD	1.35 (1.18, 1.56)	1.25 (1.20, 1.29)	0.23	1.42 (1.23, 1.62)	1.26 (1.21, 1.30)	0.09
**Intellectual disability**						
Planned CD	1.46 (1.11, 1.91)	1.46 (1.34, 1.58)	0.95	1.72 (1.31, 2.26)	1.47 (1.36, 1.60)	0.33
Intrapartum CD	1.20 (0.97, 1.51)	1.47 (1.36, 1.59)	0.10	1.45 (1.06, 1.98)	1.53 (1.42, 1.64)	0.70

© 2021 Zhang T et al. *JAMA Network Open.*

(iii) Indications for planned CD

	Pelvic disproportion		
	Yes	No	p-value
**ASD**			
Planned CD	2.26 (0.31, 16.35)	1.42 (1.33, 1.51)	0.63
**ADHD**			
Planned CD	1.47 (0.54, 3.97)	1.29 (1.24, 1.34)	0.71
**Intellectual disability**			
Planned CD	1.55 (0.21, 11.33)	1.54 (1.44, 1.67)	0.98

(iv) Indications for intrapartum CD

	Fetal distress			Failed induction			Placental disorders		
	Yes	No	p-value	Yes	No	p-value	Yes	No	p-value
**ASD**									
Intrapartum CD	1.31 (1.19, 1.45)	1.33 (1.24, 1.43)	0.91	0,96 (0.82, 1.13)	1.37 (1.29, 1.45)	**<0.001**	1.27 (0.92,1.76)	1.40 (1.33, 1.48)	0.60
**ADHD**									
Intrapartum CD	1.17 (1.10, 1.25)	1.26 (1.21, 1.32)	0.10	0.88 (0.79, 0.97)	1.24 (1.20, 1.29)	**<0.001**	1.41 (1.15, 1.73)	1.27 (1.22, 1.31)	0.32
**Intellectual disability**									
Intrapartum CD	1.44 (1,28, 1.63)	1.24 (1.13, 1.37)	**0.04**	1.03 (0.82, 1.30)	1.55 (1.44, 1.67)	**<0.001**	1.92 (1.34, 2.75)	1.51 (1.41, 1.63)	0.16

	Dystocia			Pelvic disproportion		
	Yes	No	p-value	Yes	No	p-value
**ASD**						
Intrapartum CD	1.23 (1.11, 1.36)	1.40 (1.31, 1.49)	**0.04**	0.97 (0.51, 1.84)	1.40 (1.33, 1.48)	0.23
**ADHD**						
Intrapartum CD	1.17 (1.10, 1.25)	1.26 (1.20, 1.31)	0.09	0.96 (0.64, 1.45)	1.27 (1.23, 1.32)	0.16
**Intellectual disability**						
Intrapartum CD	1.09 (0.93, 1.27)	1.74 (1.61, 1.89)	**<0.001**	2.21 (0.68, 7.16)	1.54 (1.44, 1.66)	0.58

Abbreviations: ADHD: attention-deficit/hyperactivity disorder; ASD: autism spectrum disorders; CIs: confidence intervals; CD: cesarean delivery; HR: hazard ratio.

© 2021 Zhang T et al. *JAMA Network Open.*

eTable 6. Estimated Risk of Neurodevelopmental Disorders Among Individuals Delivered by Planned and Intrapartum Cesarean Delivery Compared to Vaginally-Delivered Individuals, by Gestational Age

	**37 weeks** HR (95% CIs)	**38 weeks** HR (95% CIs)	**39 weeks** HR (95% CIs)	**40 weeks** HR (95% CIs)	**41 weeks** HR (95% CIs)	**≥ 42 weeks** HR (95% CIs)
**Any neurodevelopmental disorder**[a]						
Planned CD[b]	**1.14 (1.03, 1.27)**	**1.09 (1.02, 1.15)**	**1.22 (1.12, 1.33)**	**1.27 (1.09, 1.47)**	**1.30 (1.07, 1.58)**	1.16 (0.94, 1.42)
Intrapartum CD[c]	1.11 (0.97, 1.26)	**1.18 (1.08, 1.30)**	**1.26 (1.16, 1.36)**	**1.14 (1.06, 1.23)**	**1.10 (1.02, 1.19)**	1.00 (0.91, 1.10)
**Autism spectrum disorders**						
Planned CD[b]	1.17 (0.95, 1.45)	1.09 (0.97, 1.22)	**1.30 (1.10, 1.53)**	1.24 (0.92, 1.65)	1.30 (0.90, 1.88)	1.27 (0.88, 1.84)
Intrapartum CD[c]	1.25 (0.99, 1.60)	1.14 (0.95, 1.37)	**1.33 (1.13, 1.55)**	1.08 (0.93, 1.24)	1.15 (1.00, 1.33)	1.10 (0.93, 1.29)
**Attention-deficit/hyperactivity disorder**						
Planned CD[b]	**1.18 (1.04, 1.33)**	**1.09 (1.02, 1.17)**	**1.17 (1.06, 1.30)**	**1.28 (1.07, 1.52)**	1.17 (0.92, 1.49)	1.17 (0.92, 1.50)
Intrapartum CD[c]	1.15 (0.99, 1.33)	**1.17 (1.05, 1.31)**	**1.25 (1.13, 1.38)**	**1.12 (1.03, 1.23)**	**1.10 (1.01, 1.20)**	0.94 (0.84, 1.05)
**Intellectual disability**						
Planned CD[b]	**1.38 (1.06, 1.79)**	1.08 (0.92, 1.26)	**1.43 (1.15, 1.78)**	**1.71 (1.17, 2.50)**	**2.01 (1.29, 3.13)**	**1.80 (1.13, 2.85)**
Intrapartum CD[c]	**1.40 (1.02, 1.90)**	**1.42 (1.14, 1.78)**	**1.67 (1.37, 2.04)**	**1.72 (1.43, 2.06)**	**1.35 (1.10, 1.66)**	**1.31 (1.05, 1.65)**

Abbreviations: CIs: confidence intervals; CD: cesarean delivery; HR: hazard ratio.

[a] Any neurodevelopmental disorder includes any of the following diagnoses: attention-deficit/hyperactivity disorder, autism spectrum disorders, intellectual disability, tic disorders, communication disorders, and learning disorders.

[b] Adjusted for offspring's sex and year of birth, gestational age, age of mother and father, parity, mother's highest education level, maternal smoking during pregnancy, maternal and paternal history of psychiatric disorders, maternal hypertension, maternal diabetes, maternal infections during pregnancy, fetal malpresentation, large for gestational age, polyhydramnios, oligohydramnios, pre-eclampsia, and pelvic disproportion.

[c] Adjusted for offspring's sex and year of birth, gestational age, age of mother and father, parity, mother's highest education level, maternal smoking during pregnancy, maternal and paternal history of psychiatric disorders, maternal hypertension, maternal diabetes, maternal infections during pregnancy, fetal malpresentation, large for gestational age, polyhydramnios, oligohydramnios, pre-eclampsia, pelvic disproportion, placenta disorders, dystocia, failed induction, and fetal distress.

© 2021 Zhang T et al. *JAMA Network Open.*

eTable 7. Estimated Risk of Autism Spectrum Disorder With and Without Intellectual Disability Among Individuals Delivered by Planned and Intrapartum Cesarean Delivery, Compared to Vaginally-Delivered Individuals

	No. of individuals	Unadjusted model, HR (95% CIs)	Partially adjusted,[a] HR (95% CIs)	Fully adjusted,[b] HR (95% CIs)
**ASD with ID**				
Planned CD	220	1.12 (0.97, 1.28)	1.01 (0.87, 1.17)	1.02 (0.88, 1.19)
Intrapartum CD	273	**1.15 (1.02, 1.30)**	1.97 (0.85, 1.10)	0.97 (0.85, 1.11)
**ASD without ID**				
Planned CD	962	**1.55 (1.45, 1.66)**	**1.21 (1.13, 1.30)**	**1.21 (1.12, 1.30)**
Intrapartum CD	1142	**1.52 (1.43, 1.61)**	**1.15 (1.07, 1.22)**	**1.15 (1.07, 1.22)**

Abbreviations: ASD: autism spectrum disorders; CIs: confidence intervals; CD: cesarean delivery; HR: hazard ratio; ID: intellectual disability.

[a] Adjusted for offspring's sex and year of birth, gestational age, age of mother and father, parity, mother's highest education level, maternal smoking during pregnancy, and maternal and paternal history of psychiatric disorders.
[b] In addition to variables adjusted in a, b further adjusted for maternal hypertension, maternal diabetes, maternal infections during pregnancy, fetal malpresentation, large for gestational age, polyhydramnios, oligohydramnios, and pre-eclampsia.

© 2021 Zhang T et al. *JAMA Network Open.*

eReferences

1. Marsal K, Persson PH, Larsen T, et al. Intrauterine growth curves based on ultrasonically estimated foetal weights. Acta Paediatr. 1996;85(7):843-848.
2. Ruck C, Larsson KJ, Lind K, et al. Validity and reliability of chronic tic disorder and obsessive-compulsive disorder diagnoses in the Swedish National Patient Register. BMJ Open. 2015;5(6).

© 2021 Zhang T et al. *JAMA Network Open.*
